# A complex bearing TSPO PIGA ligand coordinated to the [Au(PEt_3_)]^+^ pharmacophore is highly cytotoxic against ovarian cancer cells

**DOI:** 10.1007/s10534-023-00496-8

**Published:** 2023-03-04

**Authors:** Lorenzo Chiaverini, Emma Baglini, Michele Mannelli, Valeria Poggetti, Federico Da Settimo, Sabrina Taliani, Tania Gamberi, Elisabetta Barresi, Diego La Mendola, Tiziano Marzo

**Affiliations:** 1 Department of Pharmacy, University of Pisa, Via Bonanno Pisano 6, 56126 Pisa, Italy; 2 Department of Experimental and Clinical Biomedical Sciences “Mario Serio”, University of Florence, Viale GB Morgagni 50, 50134 Florence, Italy; 3 University Consortium for Research in the Chemistry of Metal Ions in Biological Systems (CIRCMSB), Via Celso Ulpiani 27, 70126 Bari, Italy

**Keywords:** Gold complexes, TSPO, Thioredoxin reductase, Ovarian cancer

## Abstract

Auranofin ([1-(thio-κS)-β-d-glucopyranose-2,3,4,6-tetraacetato](triethylphosphine)-gold) is a leading gold-based drug clinically used to treat arthritis. In the last years, it entered various drug reprofiling programs, and it has been found promising against various forms of tumor, including ovarian cancer. Evidence showed as its antiproliferative profile mainly depends on the inhibition of thioredoxin reductase (TrxR), being this mitochondrial system its main target. In this context, we report here the synthesis and biological evaluation of a novel complex designed as auranofin analogue obtained through the conjugation of a phenylindolylglyoxylamide ligand (which belongs to the so-called PIGA TSPO ligand family) with the auranofin-derived cationic fragment [Au(PEt_3_)]^+^. This complex is characterized by two parts. The phenylindolylglyoxylamide moiety, owing to its high affinity for TSPO (in the low nM range) should drive the compound to target mitochondria, whereas the [Au(PEt_3_)]^+^ cation is the actual anticancer-active molecular fragment. Overall, we wanted to offer the proof-of-concept that by coupling PIGA ligands to anticancer gold active moieties, it is possible to preserve and even improve anticancer effects, opening the avenue to a reliable approach for targeted therapy.

**Supplementary Information:**

The online version of this article contains supplementary material available 10.1007/s10534-023-00496-8.

## Introduction

**Fig. 1:**
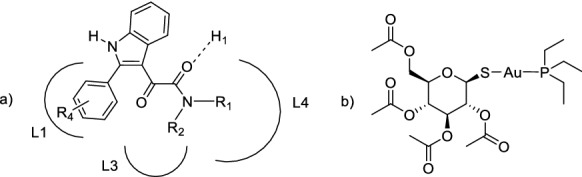
**a** *N*,*N*-dialkyl(2-phenylindol-3-yl)glyoxylamides PIGAs in the TSPO pharmacophore/topological model; **b** chemical structure of auranofin

In the last years, gold complexes have attracted growing attention for medicinal applications (Nardon et al. 2014; Guarra et al. 2018; Balfourier et al. 2020; Lu et al. 2022). Although chrysotherapy has been used for centuries for various indications including skin infections and seizures (Wang et al. 2012), one of the first modern evidence, concerning the medicinal role of gold, can be dated back to 1890 when Robert Koch discovered that gold cyanide was effective against the *tuberculosis bacillus *in vitro (Balfourier et al. 2020). More recently, the approval of the Au(I) compound auranofin (AF hereafter) for the treatment of rheumatoid arthritis has contributed to stimulate increasing interest on the medicinal properties of gold-based molecules (Ott 2009; Nobili et al. 2010; Barry and Sadler 2013; Bertrand and Casini 2014; Massai et al. 2021; Cirri et al. 2022). Accordingly, several gold(I) and gold(III) compounds have been synthesized and tested for their anticancer, antimicrobial, antiviral and antiparasitic properties (Ott 2009; Massai et al. 2017; Yeo et al. 2018; Guarra et al. 2018; Bian et al. 2019; Marzo and Messori 2020; Maydaniuk et al. 2021; Chiaverini et al. 2022; Liu et al. 2022). Among them, particularly promising appear the analogues of AF bearing different ligands in place of the thiosugar. Several compounds belonging to this family resulted very promising for application against cancer thanks to their ability to tightly interact and coordinate the selenocysteine residue of the thioredoxin reductase (TrxR). This interaction impairs the key function of the enzyme, eventually inducing cell death (Marzo et al. 2019; Zoppi et al. 2020; Landini et al. 2020; Elie et al. 2020). AF derivatives are designed on the basis of the established concept that AF structure (Fig. 1) is characterized by the presence of a moiety acting as pharmacophore, the [Au(PEt_3_)]^+^ cation, and the thiosugar ligand linearly coordinated to the Au(I) center, that is not essential for the pharmacological activity. Rather, it is capable to improve the bioavailability profile upon oral administration (Sutton et al. 1972). In nice agreement with this view, previous studies showed that Et_3_PAuCl manifests biological properties similar to those of AF (Sutton et al. 1972). This implies that the chemical-physical features and the effects of AF can be conveniently tuned and modulated through the selective replacement of the thiosugar with ligands endowed with specific properties. This allows to control relevant parameters important for the desired pharmacological action (Marzo et al. 2017).

On the ground of these premises, we recently started to consider the chance to develop a complex in which the thiosugar is replaced with a ligand endowed with high affinity for the translocator protein (TSPO).

TSPO is a highly conserved nuclear-encoded 18 kDa protein, primarily localized in the outer mitochondrial membrane (Papadopoulos et al. 2006). Aberrant expression of TSPO has been linked to multiple diseases, including ovarian cancer (OC) (Trapani et al. 2013; Nutma et al. 2021).

Starting from 2004 (Primofiore et al. 2004; Da Settimo et al. 2008; Barresi et al. 2015, 2021) some of us developed a class of potent and selective TSPO ligands, namely the *N*,*N*-dialkyl-2-arylindol-3-ylglyoxylamides (PIGAs, Fig. 1), endowed with nanomolar/subnanomolar TSPO affinity and safe profile (Santoro et al. 2016). Specifically, many PIGA compounds have been shown to elicit marked increase in pregnenolone concentration both in vitro and in murine models (Costa et al. 2016; Santoro et al. 2016).

The strategy behind this work relies on the exploitation of TSPO overexpression that occurs in OC. Indeed, owing to the high affinity for TSPO of compounds belonging to the PIGA family, the use of these latter as ligands may allow the selective mitochondrial receptor-mediated delivery of the [Au(PEt)_3_]^+^ anticancer pharmacophore to the cancer site. In turn, the activation of such complexes is mediated by the release of [AuPEt_3_]^+^, able to inhibit the TrxR and to exert its antiproliferative activity.

**Scheme 1:**
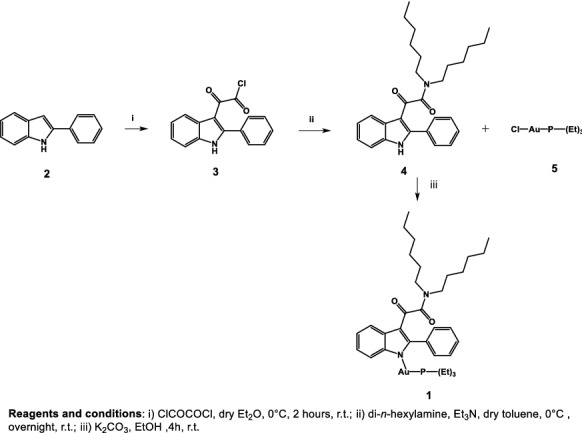
Synthesis of the target compound **1**

For the rational design of the new complex **1** (Scheme 1), we have taken into account the previously described pharmacophore/topological model for the binding of PIGAs to TSPO (Fig. 1), made up of three lipophilic pockets L1, L3, and L4, occupied by the 2-phenyl group, and the aryl/alkyl substituents on the amide nitrogen, respectively, and an H-bond donor group interacting with the amide carbonyl group (Primofiore et al. 2004; Da Settimo et al. 2008; Barresi et al. 2015). Accordingly, the indole nitrogen is suitable to be exploited for the coordination with the [Au(PEt_3_)]^+^ anticancer cationic fragment, the “true pharmacophore”, as the 1-NH is supposed not to be involved in a direct interaction with the mitochondrial target protein TSPO.

To this aim, the *N*-unsubstituted PIGA **4** (Scheme 1) (Primofiore et al. 2004) was selected as representative of the whole class, to be complexed with the [Au(PEt_3_)]^+^ fragment.

**Fig. 2:**
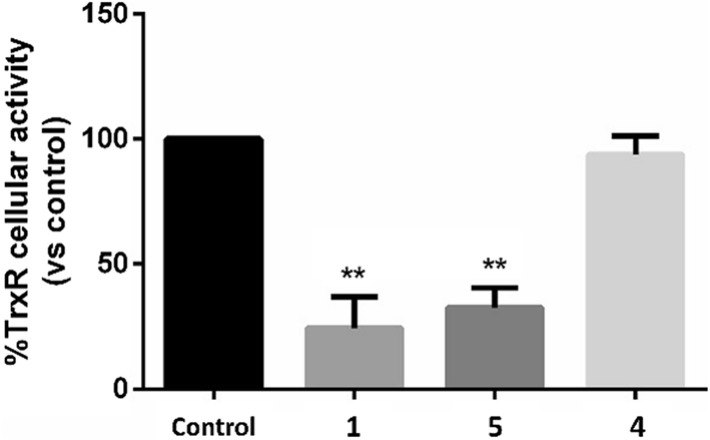
Residual % of Thioredoxin Reductase (TrxR) activity after treatment of A2780 cells for 24 h with compounds’ concentrations corresponding to their 72 h-exposure IC_50_-doses. Values are mean ± standard deviation (SD) of three biological independent experiment (**ANOVA p-value < 0.01)

The novel complex **1** (Scheme 1) was obtained and fully characterized by means of NMR and elemental determination measurements. The cytotoxic activity of complex **1** was tested on A2780 and SKOV-3 human OC cell lines and on HEK293 human embryonic kidney cell line (Table 1). In addition, TrxR activity of the complex was tested on A2780 cell line (Fig. 2) and on isolated enzyme (Table 2).

**Table 1:** Half-maximal inhibitory concentration (IC_50_) of the investigated compounds **1**, **4** and **5** after 72 h of treatment using MTT assay

Compound	Cell line- IC_50_ (µM) ± SD^a^		
	A2780	SKOV-3	HEK-293
**1**	1.47 ± 0.48	1.90 ± 0.30	9.00 ± 0.47
**4**	14.00 ± 3.77	10.3 ± 0.60	> 100
**5**	2.48 ± 0.55	2.73 ± 0.77	15.20 ± 2.14

^a^Values are mean ± standard deviation (SD) of three biological independent experiments

**Table 2:** The half-maximal effective concentration (EC_50_) values for the TrxR inhibition by compounds **1**, **4**, **5**

Compound	EC_50_ (nM)*
**1**	59.2 ± 2.8
**4**	N.D
**5**	51.1 ± 1.7

*EC_50_ mean values ± SD of three independent experiments, each carried out in triplicate. N.D. not detectable

## Materials and methods

All the reagents were provided by Merck-Sigma Aldrich and used without further purification. Et_3_PAuCl was purchased from Sigma-Aldrich (code:288225). Solvents were also used without further purification. *N,N-*di-*n*-hexyl-2-(2-phenyl-1*H*-indol-3-yl)glyoxylamide **4** was synthesized as previously described (Primofiore et al. 2004). The obtained product was stored at − 20 °C. NMR spectra were recorded at 293 K on a Bruker Avance II 400 MHz; chemical shifts (expressed in parts per million, ppm) were referenced to solvent residual peaks. Elemental analysis was performed using a vario MICRO V4.0.10 Elementar Analysensysteme GmbH.

RPMI 1640 cell culture medium, fetal bovine serum (FBS), and phosphate-buffered saline (PBS) were obtained from Euroclone (Milan, Italy). Thiazolyl Blue Tetrazolium Bromide (MTT) was obtained from Merck-Sigma Aldrich.

### Preparation of [N,N-di-n-hexyl-2-(2-phenyl-1-indol-kN-3-yl)glyoxylamido]-AuPEt_3_ 1.

In a 25 mL flask were added 39 mg (0.090 mmol) of *N,N-*di-*n*-hexyl-2-(2-phenyl-1*H*-indol-3-yl)glyoxylamide, 3.00 mL of EtOH and 16 mg (0.12 mmol) of K_2_CO_3_. The reaction mixture was stirred at room temperature for 10 min and then transferred to a schlenk tube, containing 32 mg (0.090 mmol) of Et_3_PAuCl, in which 3 vacuum-nitrogen cycles had previously been performed. After 4 h under magnetic stirring, the suspension was filtered on celite, and the filtrate removed under reduced pressure. A light brown powder was obtained which was washed with Et_2_O. The product was finally dried under reduced pressure, obtaining 45 mg of [*N*,*N*-di-*n*-hexyl-2-(2-phenyl-1-indol-kN-3-yl)glyoxylamido]-AuPEt_2_ (0.0603 mmol; yield 67%).

^1^H-NMR (DMSO-d_6_, mixture of conformational isomers): 8.02 (s, 1H); 7.62–7.57 (m, 3H); 7.40–7.38 (m, 3H); 7.16–7.09 (m, 2H); 3.00 (bs, 2H); 2.86 (bs, 2H); 1.89–1.81 (m, 6H); 1.41–0.96 (m, 25H); 0.87 (t, 3H, J = 6.9 Hz); 0.76 (t, 3H, J = 7.0 Hz).

^31^P{^1^H}-NMR (DMSO-d_6_): 32.32.

Elemental Analysis (CHN): C_34_H_50_AuN_2_O_2_P·0.5KCl; required: C = 52.09; H = 6.43; N = 3.57. Found: C = 51.56; H = 6.34; N = 3.48.

### Stability study

The stability of [*N*,*N*-di-*n*-hexyl-2-(2-phenyl-1-indol-*kN*-3-yl)glyoxylamido]-AuPEt_2_ was assessed by ^31^P{^1^H}-NMR experiments recorded UV–Vis spectra at increasing time intervals. A small quantity of the compound was solubilized in DMSO-d_6_ and NMR spectra were recorded after 2 h and 24 h. Uv–Vis spectra were recorded on a PerkinElmer Lambda 25 Uv–Vis spectrometer.

### Cell culture

A2780 human ovarian cancer cell line was purchased from the European Collection of Authenticated Cell Cultures (ECACC, a part of Public Health England) (Lot Nº 13J012, Sigma Aldrich), SKOV-3 human ovarian cancer cell line was gifted from Prof. Enrico Mini, University of Florence, Italy, and HEK (human embryonic kidney) cells were gifted from Prof. Letizia Taddei, University of Florence, Italy. Cells were grown in RPMI1640 medium supplemented with 10% FBS, 1% glutamine and 1% antibiotics at 37ºC and sub-cultured twice weekly. Split 1: 5 (3–6 × 10^4^ cells per mL).

### Cell viability assay

Cell viability was assessed using MTT [3-(4,5-dimethylthiazol-2-yl)-2,5-diphenyltetrazolium bromide] assay. Briefly, 1 × 10^4^ exponentially growing A2780 cells were seeded in 96 well-microplates for 24 h, then treated with tested compounds **1, 4** and** 5** concentrations ranging from 0.003 to 100 µM and incubated for 72 h at 37 °C in a humidified incubator. On the day of the test, cells were treated with 0.5 mg/ml MTT for 1 h at 37ºC. Following precipitation, blue formazan was dissolved in DMSO, and optical density was read at 595 nm in a microplate reader interfaced with Microplate Manager/PV version 4.0 software (BioRad Laboratories). From Absorbance measurements, half-maximal inhibitory concentration (IC_50_) values of each compound were calculated by using GraphPad Prism Software 6.0.

The effects of these calculated 72-h exposure IC_50_ doses on A2780 cell viability were also evaluated using an MTT time course assay at 24, 48, and 72 h of drug exposure. The experimental protocol described above was applied. All MTT experiments were performed in triplicate (biological replicates) and in each assay all gold compound concentrations were tested in triplicate (technical replicates).

### Thioredoxin reductase activity assay

The total thioredoxin reductase (TrxR) activity in cells was measured by using a commercial colorimetric assay kit (Sigma Aldrich CS0170) based on the reduction of 5,5’-dithiobis (2-nitrobenzoic) acid (DTNB) with NADPH to 5-thio-2 nitrobenzoic acid (TNB) at 412 nm. Since several enzymes present in biological sample can reduce DTNB, the kit also contains an inhibitor solution of mammalian thioredoxin reductase. This inhibitor allows determining the reduction of DTNB due only to TrxR activity. A2780 cells were treated for 24 h with concentrations corresponding to their 72 h-exposure IC_50_-doses and then lysed with RIPA buffer (50 mM Tris–HCl pH 7.0, 1% (v/v) NP-40, 150 mM NaCl, 2 mM ethylene glycol bis(2-aminoethylether)tetra-acetic acid, 100 mM NaF) supplemented with a cocktail of protease inhibitors. The protein concentrations in the cell lysates were determined by Bradford protein assay kit (Bio-Rad Laboratories) according to the manufacturer’s instructions and then 30 µg of proteins were used for the assay. Results were normalized to the cellular protein content. The experiments were performed in triplicate (three independent biological replicates). The statistical analysis was carried out using one-way ANOVA test followed by Tukey’s multiple comparisons test using Graphpad Prism software v 6.0. P-value p < 0.05 was considered statistically significant.

### Thioredoxin reductase inhibition “in vitro”

The half-maximal effective concentration (EC_50_) values for TrxR inhibition by tested compounds (**1**, **4** and **5**) were determined measuring the ability of the enzyme to directly reduce DTNB in the presence of NADPH. Rat liver TrxR (SigmaAldrich T9698) was diluted in 0.1 M potassium phosphate buffer pH 7.0 to a concentration of 60 nM (2U/mL). Aliquots of 25 µl of this enzyme solution were preincubated, for 5 min at 25 °C, with 25 µl of a solution containing 0.1 M potassium phosphate buffer pH 7.0, 5 mM EDTA, 0.25 mM NADPH and different concentrations of the gold complexes (from 1 mM to 1 nM). Afterwards, the reaction was started with 1 mM DTNB and monitored spectrophotometrically at 412 nm, for about 10 min. The non-interference of the gold compounds with assay components was confirmed by negative control experiments with enzyme free solution. The EC_50_ values, calculated by using GraphPad Prism Software 6.0, were reported as means ± SD of three independent experiments, each carried out in triplicate.

## Results and discussion

The synthetic procedure used for the preparation of *N,N-*di-*n*-hexyl-2-(2-phenyl-1-indol-*kN*-3-yl)glyoxylamide-AuP(Et)_2_ **1** is outlined in Scheme 1 and involved the acylation of the 2-phenylindole **2**, commercially available, with oxalyl chloride, in anhydrous ethyl ether, at room temperature, to obtain the corresponding 2-phenylindolylglyoxylyl chloride **3**, which was allowed to react at room temperature with di-*n*-hexylamine in the presence of triethylamine in dry toluene solution, yielding derivative **4** (Primofiore et al. 2004). To a solution of **4** in EtOH, K_2_CO_3_ was added and the resulting mixture was reacted at room temperature for 10 min. Subsequently, Et_3_PAuCl **5** was added, and the resulting mixture was left under stirring at room temperature for further 4 h. Then, the solid was filtered off and the filtrate was evaporated under reduced pressure, obtaining a light brown powder.

The NMR results confirmed the obtainment of the desired complex **1** (Figs. S1–S4). In particular, an highfield shift of the signal in the ^31^P{^1^H}-NMR spectrum of **1** (32.2 ppm) with respect to that of precursor **5** (33.87 ppm) (Figs. S1–S2) can be observed. Such results are in line with that reported in literature, where an highfield shift occurs when the chloride ligand of **5** is replaced by a nitrogen donor ligand (Weinstock et al. 1974). Further confirmation of the complex formation is provided by comparison of the ^1^H-NMR spectra recorded on compound **1** and the uncomplexed TSPO ligand **4**. The signals accounting for the aromatic protons in **1** are shifted in comparison to those of the free ligand **4** (Figs. S3–S4). In addition, in the **1** ^1^H-NMR spectrum, the multiplets ranging from 1.89 to 1.81 ppm and 1.10–0.9 ppm, accounting for the CH_2_ and CH_3_ of the triethylphosphine ligand coordinated to gold (I) respectively can be observed (Marzo et al. 2017).

Interestingly, ^31^P NMR spectra recorded at increasing time intervals in presence of organic solvent (DMSO), reveal **1** as highly stable in these conditions, similarly to AF and Et_3_PAuCl (Marzo et al. 2017). Indeed, no significant changes in the spectra occur (Fig. S5). The substantial stability, even in presence of cell culture medium, was independently confirmed through UV–Vis experiments (Fig. S6). After 24 h of incubation, only minor changes in the profiles were detected. To assess whether these small changes were attributable to partial ligand release, we also recorded the UV–Vis spectrum of the uncomplexed ligand **4** (Fig. S7). From the comparison, it was noticed a ligand release -despite quite limited- upon incubation in water media in physiological-like conditions.

The antiproliferative properties of the new complex **1** was investigated in vitro toward A2780 human ovarian cancer cells and compared with those of uncomplexed-ligand **4** and Et_3_PAuCl **5**. As displayed in Table 1, compounds **1** and **5** produce potent growth inhibition effects with IC_50_ in the low micromolar range, being **1** the most active one (1.47 µM), with a two-fold gain in potency with respect to the parent compound **5**. The result obtained for Et_3_PAuCl **5** (2.48 µM) is consistent with that previously obtained in the same cell line and recently published (Landini et al. 2020). On the other hand, **4** exhibited a moderate effect with a cytotoxic activity about tenfold less than **1** (Table 1).

The antiproliferative activity was also assessed on another human OC cell line (i.e., SKOV-3), confirming the growth inhibition properties of compounds, that is the higher effectiveness of compound **1** (1.90 µM) with respect to the parent compound** 5** (2.73 µM), and the moderate activity of **4** (Table 1). Remarkably, measuring the antiproliferative effects on a non-malignant cell line, HEK293 human embryonic kidney (Table 1), the IC_50_ values were found to be higher (about sixfold for both compounds **1** and **5**), suggesting a selectivity towards cancer cells.

Currently, the thioredoxin reductase (TrxR) enzyme is recognized as the main target of gold-based compounds (Bindoli et al. 2009; Zhang et al. 2019). Therefore, we evaluated the ability of **1**, **4** and **5** to inhibit TrxR (Fig. 2). A2780 cancer cells were treated for 24 h with compounds’ concentration corresponding to their 72-h-exposure IC_50_ dose to avoid the cell death. Indeed, as demonstrated by MTT time course experiments, after 24 h of exposure, the viability of treated cells was comparable to controls (Fig. S8). After 24 h of treatment, A2780 cells exposed to **1** and **5** displayed a residual TrxR activity of only 24% and 32%, respectively, compared to control cells, whereas **4** did not induce a statistically significant decrease of enzyme activity (Fig. 2).

Next, to confirm that the observed decrease of TrxR activity in treated cancer cells could be due to a direct inhibition of the enzyme, we comparatively quantified the inhibitory potency of compound **1** and **5** on the isolated TrxR enzyme. Also compound **4** was tested, showing no detectable activity. The resulting half-maximal effective concentration (EC_50_) values for enzyme inhibition are reported in Table 2. Overall, both compound **1** and **5** proved to be effective TrxR inhibitors with EC_50_ values falling in the low nanomolar range (Table 2).

These results suggest a correlation between the antiproliferative properties of **1** and **5** and their inhibitory ability towards TrxR, highlighting **1** as the most active and confirming our working hypotheses that conjugation of the anticancer-active molecular fragment [Au(PEt_3_)]^+^ cation with a TSPO ligand targeting mitochondria could retain/improve anticancer effects, thus paving the way to a reliable approach for targeted therapy (Marzo et al. 2017).

## Conclusions

In this work, a novel Au(I) complex **1** that is an analogue of AF featuring the replacement of the thiosugar with a TSPO-ligand belonging to the class of PIGAs was prepared and characterized. Similarly to AF and Et_3_PAuCl, it shows a high stability in DMSO. Notably, the novel complex **1** manifested a fully improved anticancer activity towards two representative models of human OCs (i.e. A2780 line and SKOV-3) and a far lower cytotoxicity toward a normal cell line (i.e. HEK293), offering -to the best of our knowledge- for the first time the proof-of-concept that the conjugation of the active gold fragment [Au(PEt_3_)]^+^ with PIGAs may represent a reliable strategy to develop potent anticancer gold-based complexes combined with the possibility to improve mitochondrial delivery.

### Supplementary Information

Below is the link to the electronic supplementary material.

Electronic supplementary material 1 (DOCX 483 kb)
