# Supplementary material for: A complex bearing TSPO PIGA ligand coordinated to the [Au(PEt3)]+ pharmacophore is highly cytotoxic against ovarian cancer cells
Source: Biometals. 2023 Mar 4;36(5):961–8. doi: 10.1007/s10534-023-00496-8 (PMC10545567; doi:10.1007/s10534-023-00496-8)
Supplement: Supplementary file 1 — Electronic supplementary material 1 (DOCX 483 kb) [file 10534_2023_496_MOESM1_ESM.docx]

**Supporting Information**

A complex bearing a non-cytotoxic TSPO PIGA ligand coordinated to the [Au(PEt_3_)]^+^ pharmacophore is highly cytotoxic against ovarian cancer cells.

Lorenzo Chiaverini,^[a]§^ Emma Baglini,^[a]§^ Michele Mannelli,^[b]^ Valeria Poggetti,^[a]^ Federico Da Settimo,^[a]^ Sabrina Taliani,^[a]^ Tania Gamberi,^[b]*^ Elisabetta Barresi,^[a]*^ Diego La Mendola,^[a,c]^ Tiziano Marzo.^[a,c]^

[a] Department of Pharmacy, University of Pisa, Via Bonanno Pisano 6, 56126, Pisa, Italy. E-mail: elisabetta.barresi@unipi.it.

[b] Department of Experimental and Clinical Biomedical Sciences “Mario Serio”, University of Florence, Viale GB Morgagni 50, 50134, Firenze, Italy.

[c] University Consortium for Research in the Chemistry of Metal ions in Biological Systems (CIRCMSB), Via Celso Ulpiani 27, 70126, Bari, Italy.

^§^These authors have contributed equally to this work.

**Contents**

**NMR Spectra2**

**^31^P{^1^H} NMR** *[N,N-di-n-hexyl-2-(2-phenyl-1-indol-kN-3-yl)glyoxylamido]AuPEt₃*2

**^31^P{^1^H} NMR** Et_3_PAuCl.3

**^1^H-NMR** *[N,N-di-n-hexyl-2-(2-phenyl-1-indol-kN-3-yl)glyoxylamido]AuPEt₃*4

**^1^H-NMR** *[N,N-di-n-hexyl-2-(2-phenyl-1H-indol-3-yl)glyoxylamide* 6

**^31^P{^1^H} NMR** Stability Study.6

**UV-Vis experiments**…………………………………………………………………………………………………………………………… 7

**Cell viability assay**…………………………………………………………………………………………………………………............. 8

**NMR Spectra**

**
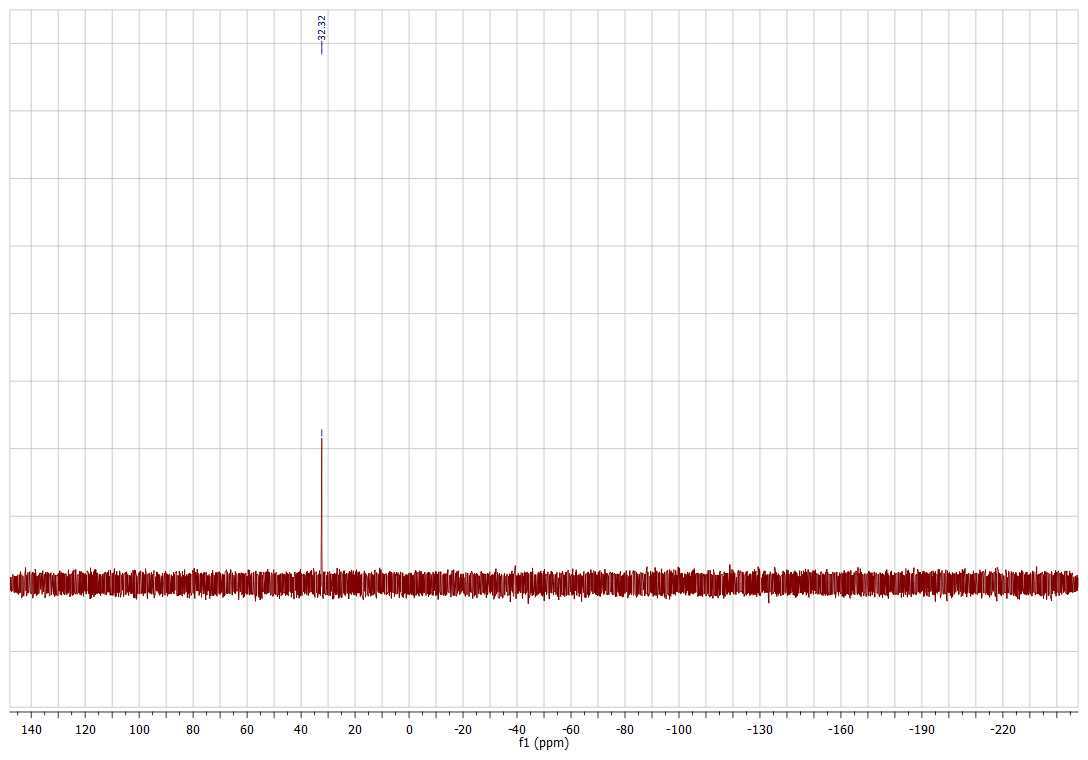
**

**Fig.S1.** *[N,N-di-n-hexyl-2-(2-phenyl-1-indol-kN-3-yl)glyoxylamido]AuPEt₃* **1**. ^31^P{^1^H}NMR (160 MHz; DMSO-d_6_) δ: 32.32.

**
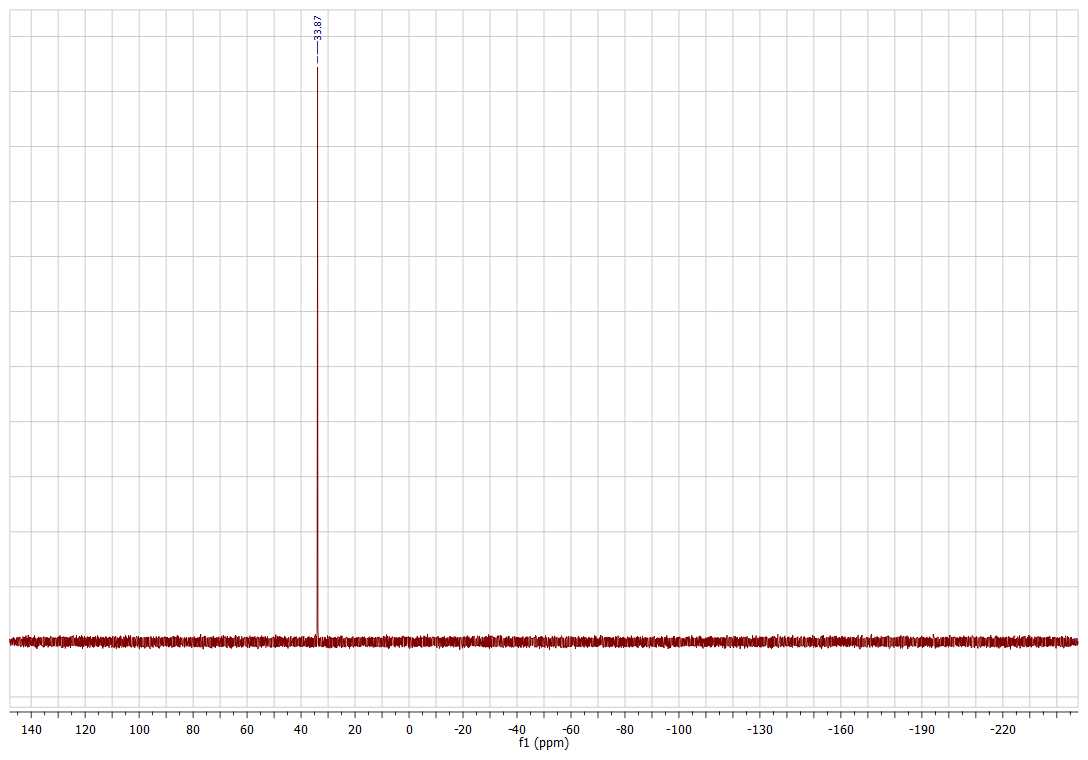
**

**Fig. S2.** *Et_3_PAuCl* ***5****.* ^31^P{^1^H}NMR (160 MHz; DMSO-d_6_) δ: 33.87.

**
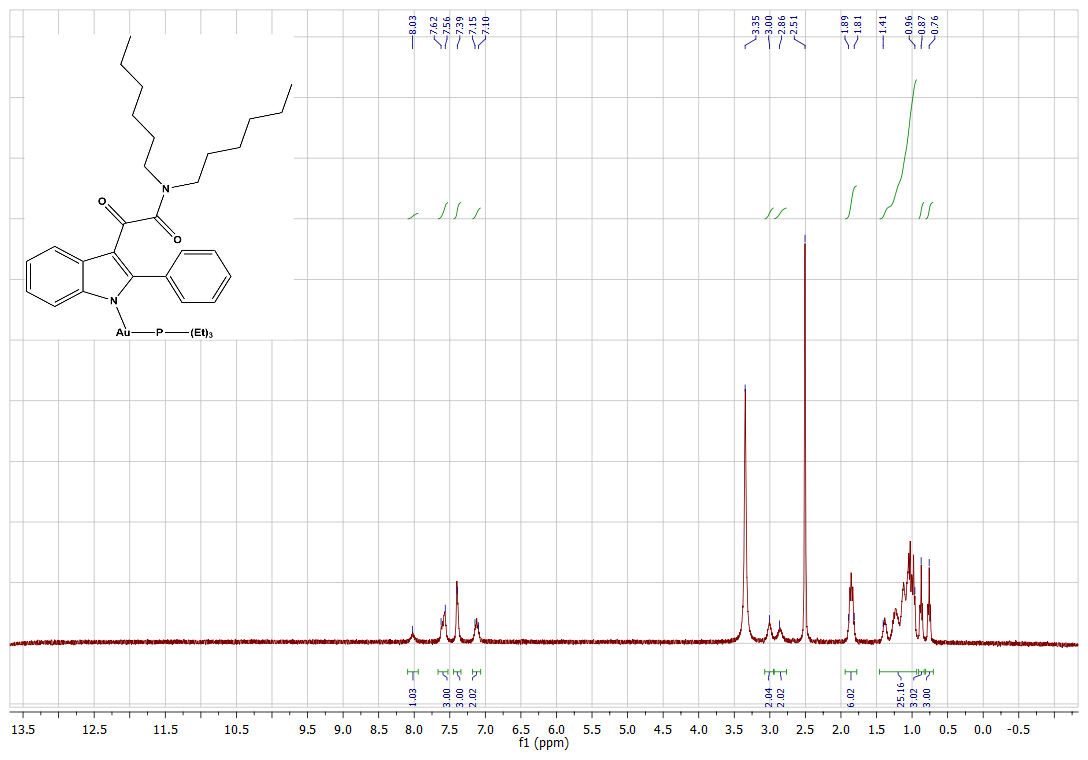
**

**Fig.S3.** *[N,N-di-n-hexyl-2-(2-phenyl-1-indol-kN-3-yl)glyoxylamido]AuPEt₃* **1**. ^1^H-NMR (400 MHz; DMSO-d_6_, mixture of conformational isomers) δ: 8.02 (bs, 1H); 7.62-7.57 (m, 3H); 7.40-7.38 (m, 3H); 7.16-7.09 (m, 2H); 3.00 (bs, 2H); 2.86 (bs, 2H); 1.89-1.81 (m, 6H); 1.41-0.96 (m, 25H); 0.87 (t, 3H, J=6.9 Hz); 0.76 (t, 3H, J=7.0 Hz).


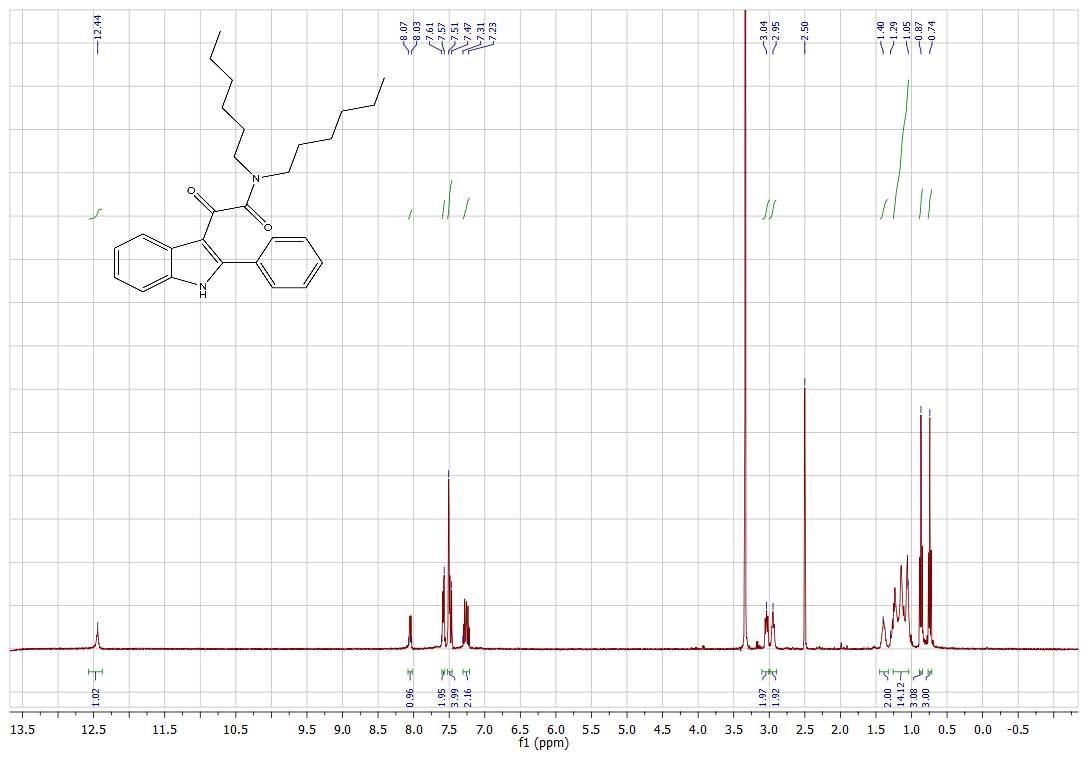


**Fig.S4.** *N,N-di-n-hexyl-2-(2-phenyl-1H-indol-3-yl)glyoxylamide* **4**. ^1^H-NMR (400 MHz; DMSO-d_6_, mixture of conformational isomers) δ: 12.44 (bs, 1H, NH); 8.07 (bs, 1H); 7.61-7.57 (m, 2H); 7.51-7.47 (m, 4H); 7.31-7.23 (m, 2H); 3.04 (bs, 2H); 2.95 (bs, 2H); 1.40 (m, 2H); 1.29-1.05 (m, 14H); 0.87 (t, 3H, J=6.9 Hz); 0.74 (t, 3H, J=7.0 Hz).


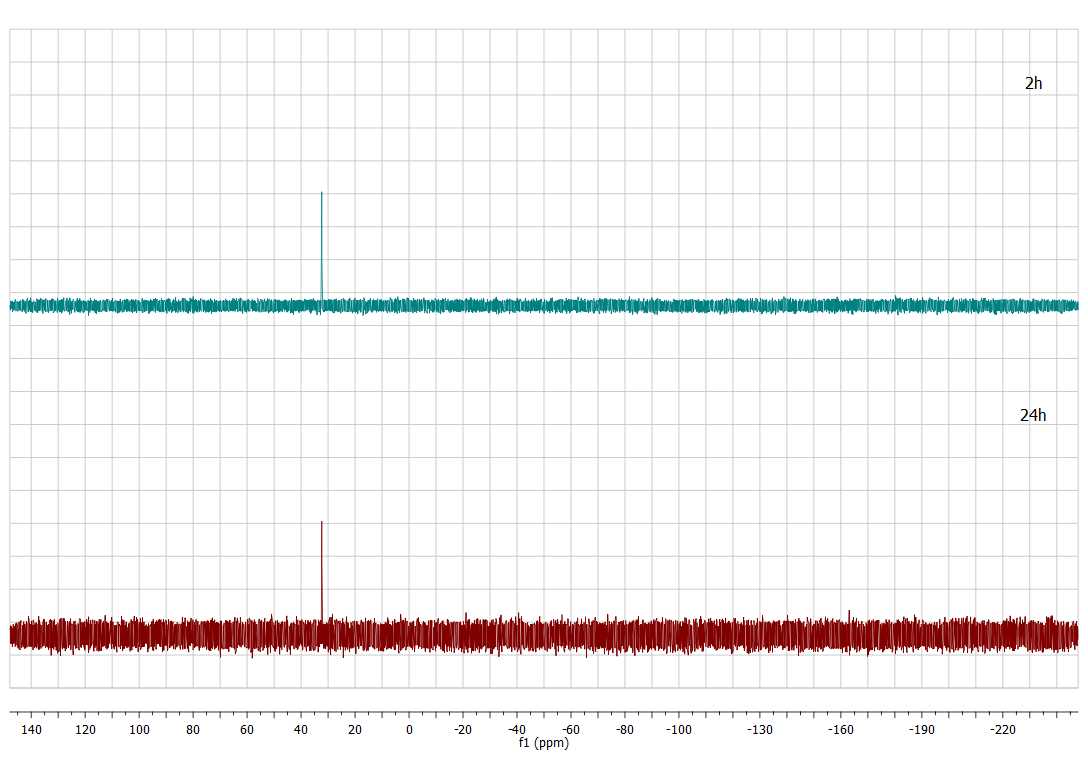


**Fig.S5.** *[N,N-di-n-hexyl-2-(2-phenyl-1-indol-kN-3-yl)glyoxylamido]AuPEt₃* **1**. Stability study; ^31^P{^1^H}NMR (160 MHz; DMSO-d_6_) δ: 32.32.

**
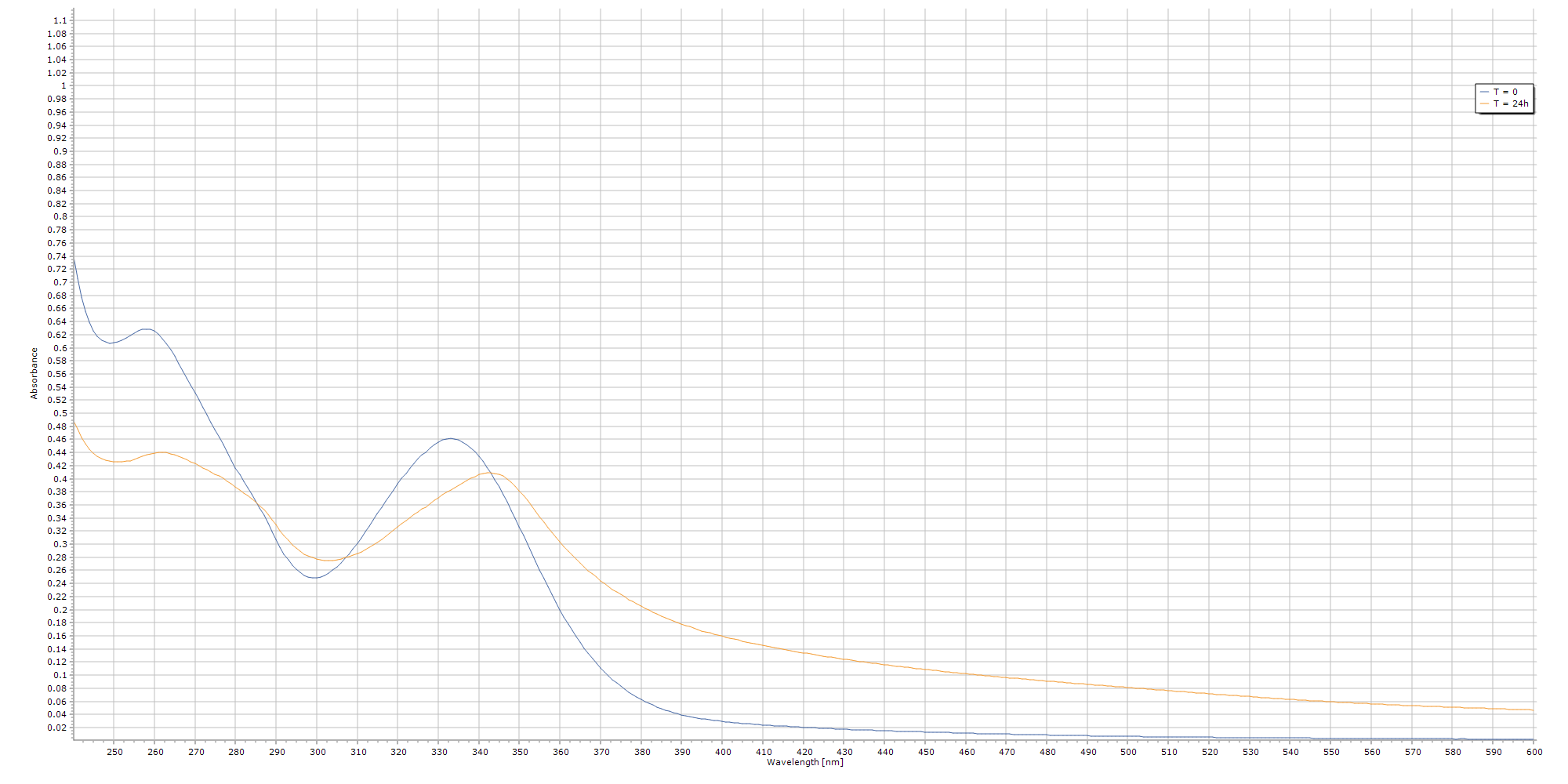
**

**Fig.S6.** Time dependent UV-Vis absorption spectra of the complex 5.29$\cdot$10^-5^ M incubated for 24 h in cell culture medium in presence of 2% of DMSO. Blue T = 0; Yellow T = 24h.

**
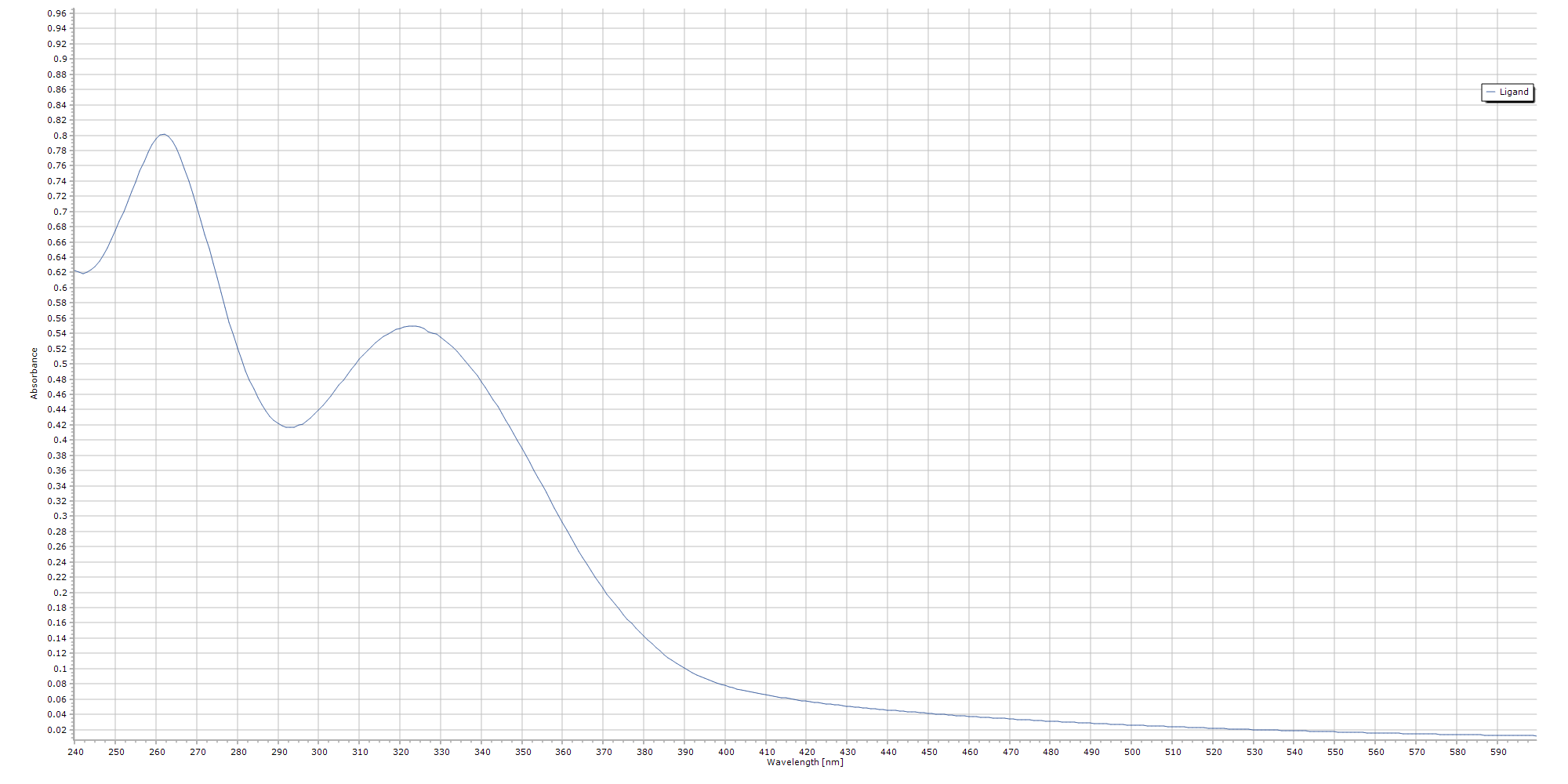
**

**Fig.S7.** UV-Vis absorption spectrum of the uncomplexed ligand 3.23$\cdot$10^-5^M *N,N-*di-*n*-hexyl-2-(2-phenyl-1*H*-indol-3-yl)glyoxylamide in cell culture medium in presence of 0.5% of DMSO.

**Fig.S8** Cell viability time course assay upon gold compound treatment. Cell viability of A2780 cancer cells after 24, 48 and 72 h of treatment with the 72 h-IC_50_ doses of each gold compound using MTT assay. The experiment was performed in triplicate (biological replicates). The histogram shows the mean values and standard deviation of the percentage of treated-A2780 viable cells relative to untreated controls. The statistical analysis was carried out using one-way ANOVA test followed by Tuckey’s multiple comparisons test using Graphpad Prism v6.0 (*p<0.05; ****p<0.0001).
